# Anemia-associated smaller brain volume and sex differences: a cross-sectional study of magnetic resonance imaging in brain health checkups

**DOI:** 10.3389/fnagi.2024.1444308

**Published:** 2024-12-06

**Authors:** Naoki Omori, Manabu Ishida, Masahiro Takamura, Satoshi Abe, Atsushi Nagai

**Affiliations:** ^1^Department of Neurology, Shimane University, Izumo, Japan; ^2^ERISA Corporation, Matsue, Japan; ^3^Institutional Research Center, Fujita Health University, Toyoake, Japan

**Keywords:** anemia, dementia, limbic system, mini mental state examination, magnetic resonance imaging, principal component analysis

## Abstract

**Introduction:**

Anemia is a risk factor for dementia development. However, few studies have examined the relationship between brain volume and anemia. This study aimed to analyze the association between anemia and brain volume using magnetic resonance imaging data from brain health checkups.

**Method:**

Participants underwent brain health checkups between January 2015 and March 2022. Blood samples were collected to measure hemoglobin concentrations and mean corpuscular volumes. The modified Mini-Mental State Examination (MMSE) was used to evaluate cognitive function. Magnetic resonance images were analyzed using voxel-based Morphometry to evaluate the overall patterns of brain volume. After extracting the principal components (PCs) from PC analysis, we investigated their association with MMSE scores and anemia.

**Results:**

This study included 1,029 participants and identified principal components, representing smaller volume in the frontal lobe (PC1), and smaller volume in the limbic system to the temporal lobe (PC2). A higher PC2 score was significantly associated with a lower MMSE score. Male participants with anemia had smaller bilateral PC1 volumes and left hippocampal volumes, and female participants with anemia had smaller bilateral PC2 volumes and hippocampus volumes.

**Discussion:**

PC2 may represent the extent of disease affecting limbic system volume, such as Alzheimer’s disease. Our results suggest that anemia may be associated with smaller volumes in the limbic system, especially in women. Further studies are required to determine which type of anemia is more strongly correlated with smaller brain volumes.

## 1 Introduction

Dementia is one of the most significant diseases affecting human health. Globally, researchers are engaged in efforts to understand its pathophysiology and treatment. In recent years, molecular pathology research on Alzheimer’s disease (AD) has been intensified, leading to the development of disease-modifying therapies, such as anti-amyloid antibodies (Bard et al., 2000; Hock et al., 2003). Because the ideal time to administer these drugs is in the early stages of cognitive decline, early detection and treatment of dementia are important (Kozauer and Katz, 2013). In Japan, health-conscious adults often undergo brain health checkups using magnetic resonance imaging (MRI), known as the Brain Dock assessment, to detect progressive brain atrophy and asymptomatic cerebrovascular disease at early stages (Morita, 2019).

Anemia is a serious medical problem affecting people of all ages. The worldwide prevalence of anemia is 32.9%, with iron deficiency anemia being the most common type (Clark, 2008; Kassebaum et al., 2014). Patients undergoing treatment for chronic diseases often present with inflammation-associated anemia (Weiss et al., 2019). Persistent anemia contributes to cognitive decline and is a risk factor for dementia (Jeong et al., 2017; Kim et al., 2019; Qin et al., 2019; Wolters et al., 2019; Weiss et al., 2022). Several studies have shown that anemia is significantly associated with AD and cerebrovascular dementia (Milward et al., 1999; Shah et al., 2011; Faux et al., 2014).

However, few studies have examined the association of anemia with brain volume. In a longitudinal study of older people, lower blood hemoglobin levels were associated with smaller total intracranial and gray matter volume (Jonassaint et al., 2014). In contrast, a cross-sectional study showed that anemia was associated with smaller bilateral hippocampal volumes in middle-aged adults (Beydoun et al., 2021). Another study demonstrated that anemia was associated with overall cortical atrophy in women, with no similar association in men (Park et al., 2016). Thus, previous studies have not clarified whether anemia causes a non-selective or site-specific reduction in brain volume, and the involvement of sex differences should also be considered.

This study aimed to analyze the association between anemia and brain volume using MRI data from middle-aged and older adults who underwent Brain Dock assessments. We also aimed to include as many different brain regions as possible in the analysis, using principal component analysis (PCA) to extract the principal components (PCs) of the participants’ regional brain volumes.

## 2 Materials and methods

### 2.1 Ethical statement and data source

**Figure 1:**
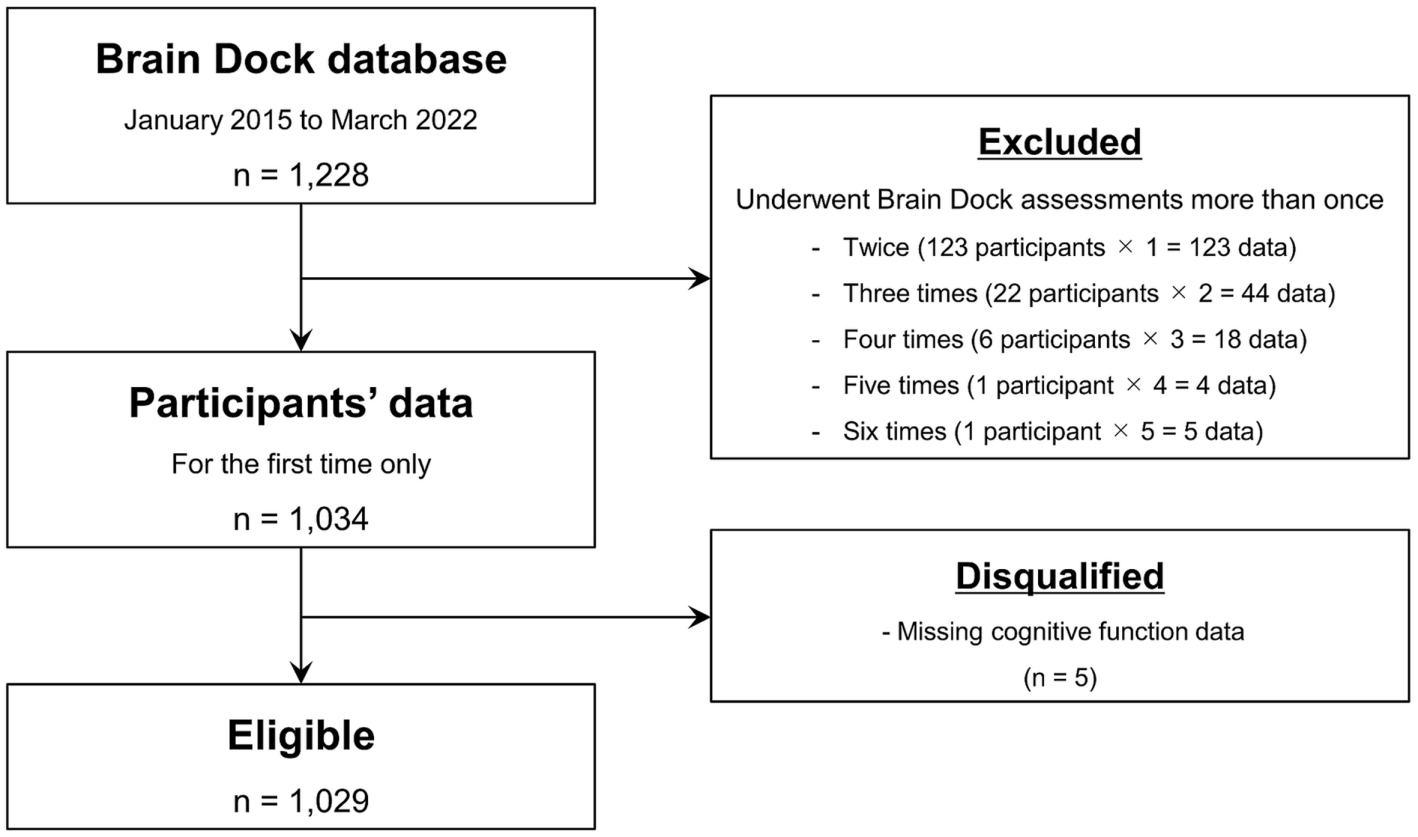
Flow chart of study sample selection.

This retrospective study was approved by the Shimane University Institutional Committee (No. 20151028-1). Written informed consent was obtained from all study participants in accordance with the Declaration of Helsinki. The participants underwent the Brain Dock assessments between January 2015 and March 2022 at the Shimane Institute of Health Science. The Brain Dock assessments using MRI and magnetic resonance angiography have been offered for brain health checkups in Japan since 1995. In addition to MRI, the assessments included medical interviews, physical examinations, mental state assessments, and laboratory-based testing. The study participants were mainly middle-aged or older adults living in the local community. Only data from the first visit were included in the analysis for those who underwent the assessment more than once. After excluding participants with missing cognitive function data, 1,029 were included in the detailed image analyses (Figure 1).

### 2.2 Clinical assessments

A medical interview and physical examination were conducted by skilled nurses. Clinical characteristics, including age, sex, body weight, height, and medication history (hypertension, diabetes mellitus, and dyslipidemia), were recorded. Body mass indices were measured as the ratios of the participants’ weight to the square of their heights (kg/m^2^). Blood samples were collected to measure the complete blood count, including hemoglobin concentration (g/dl) and mean corpuscular volume (MCV; fL). MCV was calculated by multiplying hematocrit (%) by 10 and dividing by the red blood cell count (RBC; 10^6^/μl). Nurses obtained samples in the morning after an 8–12-h overnight fast and quickly measured those using automated analyzers. The lower limit of hemoglobin was defined as 13.1 g/ml for men and 12.1 g/ml for women with one significant digit, based on the reference value of the Japan Society of Ningen Dock (Yamakado, 2016). Participants with values below these limits were classified as having amenia. MCV was classified into three groups regardless of sex: MCV < 80 fL, microcytic RBC; MCV of 81–100 fL, normocytic RBC; and MCV > 100 fL, macrocytic RBC. The modified Mini-Mental State Examination (MMSE) scale was evaluated face-to-face by an experimental occupational therapist who had been systematically trained. The MMSE scale was completed in 10–15 min. The MMSE scale is used for cognition testing and estimates cognitive function based on the MMSE score, with a maximum score of 30. The MMSE consists of 11 questions assessing the following cognitive functions: orientation to time and place, recall ability, short-term memory, arithmetic ability, comprehension, language, and basic motor skills (Folstein et al., 1975). No consistent criteria exist regarding the cut-off values of the MMSE for detecting cognitive impairment. In this study, the cut-off values were established based on previous studies (O'Bryant et al., 2008; Ciesielska et al., 2016). Cognitive normal to mild cognitive impairment was indicated by an MMSE score of 24–30, and moderate to severe cognitive impairment by a score ≤ 23.

### 2.3 Neuroimaging assessments

The laboratory MRI was changed from a Siemens 1.5 T scanner to a Philips 3.0 T scanner in January 2016 during the study period. The Siemens 1.5 T scanner was used for 108 participants, and the Philips 3.0 T scanner was used for 921 participants. Images were obtained using conventional T1-weighted, T2-weighted, and fluid-attenuated inversion recovery imaging. The acquisition parameters were as follows: repetition time, 1,800 ms; echo time, 3.7 ms; inversion time, 1,100 ms; voxel size, 1.0 × 1.0 × 1.2 mm for the 1.5 T system; repetition time, 6 ms; echo time, 2.7 ms; voxel size, 0.9 × 0.9 × 0.9 mm for the 3.0 T system.

MRI data were analyzed using Brain Anatomical Analysis using Diffeomorphic deformation (BAAD, Shiga University of Medical Science, Shiga, Japan, version 4.4.0), which is designed to support voxel-based morphometry (VBM). VBM is a sophisticated technique that standardizes variables by normalizing the brain shape through coordinate transformations and adjusting for local volumes with covariate corrections at each voxel. This method facilitates the quantitative analysis of the brain anatomy by allowing comparisons of the concentrations of brain tissue types (gray matter and white matter) to be compared across individuals. The procedural standards and methodological details of VBM have been extensively discussed in a previous publication (Kurth et al., 2015). BAAD segmented MRI T1-weighted images into gray matter, white matter, and cerebrospinal fluid and warped them into the Montreal Neurological Institute space, which was created as a new standard brain using a large series of MRI scans on healthy controls (Collins et al., 1998). The brain images reconstructed using the Montreal Neurological Institute space were further segmented into 45 anatomical volumes of interest (AVOIs) in each hemisphere based on the automated anatomical labeling atlas (Tzourio-Mazoyer et al., 2002). The AVOIs included the cerebral cortex, limbic system, and basal ganglia. The brainstem and cerebellum were excluded from analysis. Ninety AVOIs were calculated as standardized scores. Further details regarding BAAD are available in the literature (Syaifullah et al., 2020).

### 2.4 Statistical analysis

Descriptive statistics, including frequency (%), mean, standard deviation (SD), interquartile range, and median, were used to summarize the participants’ clinical characteristics, laboratory data, and MMSE scores. Categorical variables were compared using the Chi-square test. Continuous variables were tested for normality using the Kolmogorov–Smirnov test and compared using the Student’s *t*-test or Mann–Whitney U test, as appropriate. We first examined the overall patterns of brain volumes in participants using PCA with varimax rotation to define the hidden content structure in the 45 AVOIs in each hemisphere. The Kaiser–Meyer–Olkin measure of sampling adequacy (KMO > 0.7) and Bartlett’s test of sphericity (significance threshold, *p* < 0.05) were used to determine whether the data were suitable for PCA. The extraction criteria for the PCs were determined based on parallel analysis (Humphreys and Ilgen, 1969). After extracting the PCs, we interpreted them anatomically, starting with the PCs with the highest percentages of variance. The PC scores were standardized and stored for subsequent analyses. Second, the Student’s *t*-test was conducted to evaluate the association between the PC scores and MMSE scores. Since the distribution of the MMSE scores was not expected to follow a normal distribution, they were classified into two groups: normal cognitive function (MMSE score: 24–30) and cognitive impairment (MMSE score ≤ 23). Finally, an analysis of covariance (ANCOVA) was used to examine the effects of anemia on the PC scores. We included participants’ age, body mass index (BMI), history of hypertension, diabetes mellitus, dyslipidemia, and MRI magnetic field strength as covariates. The ANCOVA was stratified by sex because the degree of anemia is sex-dependent, and the participants’ sex could be a confounding factor. In addition to these models, an ANCOVA with hippocampal volume scores as the dependent variable was also performed. As MRI data collected on 1.5 T and 3.0 T scanners were mixed in this study, further analyses were limited to participants with imaging data acquired on the 3.0 T scanner to assess the consistency of the results.

All analyses were performed using Jamovi statistical software (Jamovi Project, Sydney, Australia, version 2.4.6). Statistical significance was defined as a two-sided *p* value of < 0.05.

## 3 Results

### 3.1 Baseline characteristics of the participants

The baseline characteristics of the study participants are shown in Table 1. The mean age and SD were 61.43 and 12.91 years (range: 33–87). The proportion of women was 45.87%. The mean age did not differ significantly between men and women (men: 60.92, women: 62.04, *p* = 0.164). The proportion of men taking medications for hypertension, diabetes mellitus, and dyslipidemia was significantly higher than that of women. Blood hemoglobin levels were significantly lower in women than in men. The percentage of patients with microcytic anemia (MCV < 80 fL) was higher in wemen than in men. Notably, none of the female participants with anemia had macrocytic anemia and none of the male participants with anemia had microcytic anemia. The median MMSE score was 29 points, with no significant differences between the sexes. Ninety AVOIs were measured in both hemispheres by analyzing the MRI images using BAAD. If poor-quality MRI images were taken due to body motion or gross MRI abnormalities, such as large brain tumors, infarcts, and old hemorrhages, BAAD would have difficulty analyzing them. However, we succeeded in obtaining AVOI scores for all 1,029 participants. All AVOI scores were adjusted for age and sex and were standardized. Higher scores indicated smaller brain volume.

**Table 1:** Characteristics of the participants stratified by sex.

Characteristics	Total (*n* = 1,029)	Male (*n* = 557)	Female (*n* = 472)	*p* value
Age, y, mean (SD)	61.43 (12.91)	62.04 (13.49)	60.92 (12.28)	0.164
BMI (SD)	22.77 (3.28)	23.66 (3.21)	21.70 (3.04)	<0.001
Medication				
Hypertension, *n* (%)	315/1,029 (30.61%)	190/557 (34.11%)	125/472 (26.48%)	0.008
Diabetes mellitus, *n* (%)	68/1,029 (6.61%)	47/557 (8.44%)	21/472 (4.45%)	0.012
Dyslipidemia, *n* (%)	222/1,029 (21.57%)	126/557 (22.62%)	96/472 (20.34%)	< 0.01
Laboratory data				
Hemoglobin, g/dl, mean (SD)	14.26 (1.46)	15.02 (1.31)	13.37 (1.08)	< 0.001
Anemia, *n* (%)	75/1,029 (7.29%)	26/557 (4.67%)	49/472 (10.38%)	< 0.001
MCV, *n* (%)				
< 80	12/75 (16.00%)	0/26 (0%)	12/49 (24.5%)	< 0.001
80–100	58/75 (77.33%)	21/26 (80.8%)	37/49 (75.5%)	
> 100	5/75 (6.67%)	5/26 (19.2%)	0/49 (0%)	
Cognitive function test				
MMSE score, median (interquartile)	29 (28–30)	29 (28–30)	30 (28–30)	0.111
28–30 points, *n* (%)	837/1,029 (81.34%)	457/557 (82.05%)	380/472 (80.51%)	0.630
24–27 points, *n* (%)	176/1,029 (17.10%)	93/557 (16.70%)	83/472 (17.58%)	
≤ 23 points, *n* (%)	16/1,029 (1.55%)	7/557 (1.26%)	9/472 (1.91%)	

Values represent the mean (standard deviation) for continuous variables and *n* (%) for categorical variables. The *t*-test was used to compare age, BMI, and blood hemoglobin concentration. The Mann–Whitney U test was used to compare MMSE scores. The chi-square test was used to compare group differences in the hypertension, diabetes mellitus, dyslipidemia, anemia, MCV category, and MMSE score categories. BMI, body mass index; MCV, mean corpuscular volume; MMSE, Mini-Mental State Examination; SD, standard deviation.

### 3.2 PC analysis

**Figure 2:**
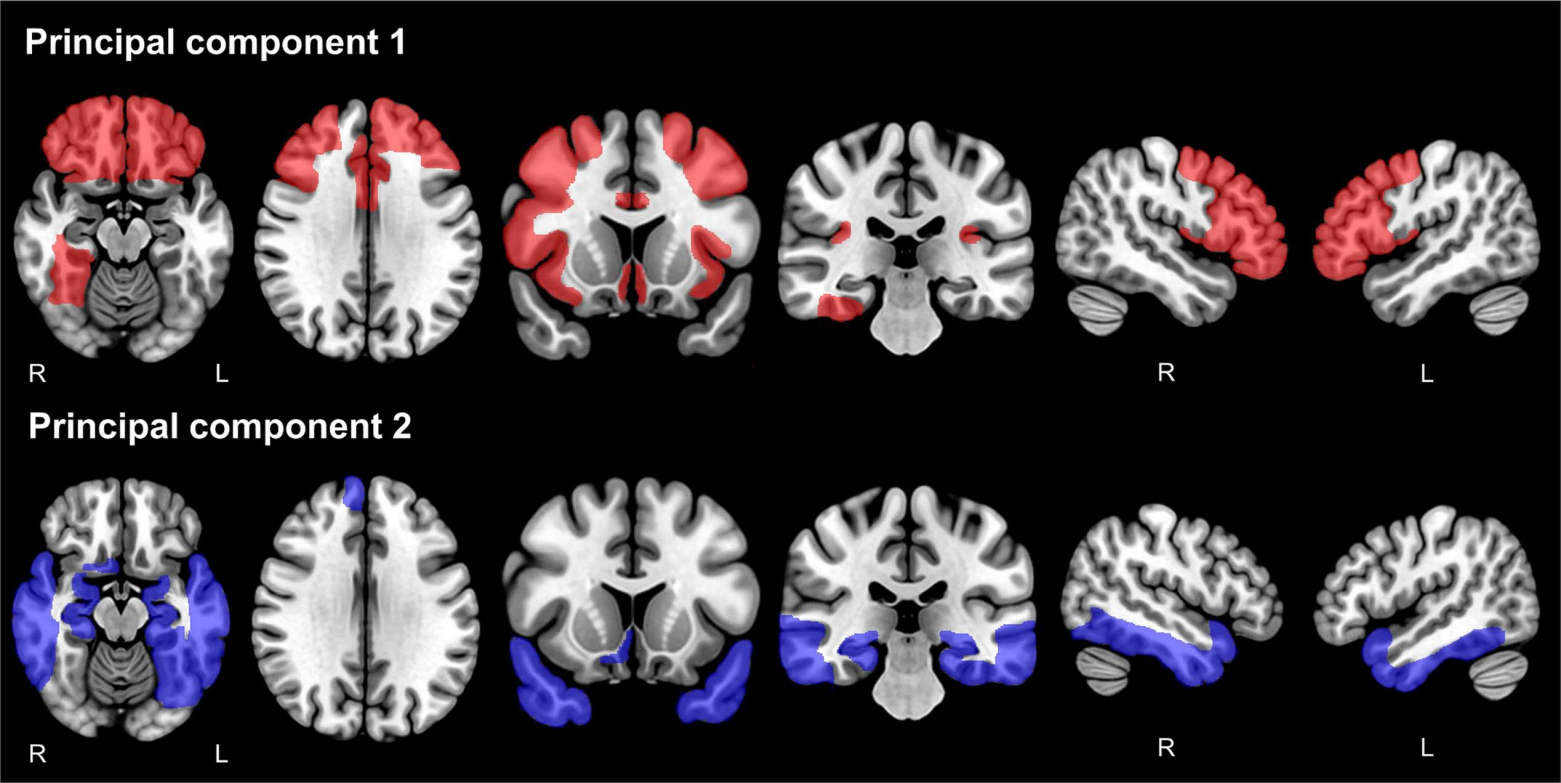
Anatomical volumes of interest with factor loadings of 0.4 or higher for principal component 1 and principal component 2. The anatomical volumes of interest with factor loadings of 0.4 or higher for PC1 are shown in red (upper panels), and for PC2, they are shown in blue (lower panels). PC, principal component.

The PCs were extracted from 45 AVOIs using PCA in each hemisphere. The evaluation of the baseline data was associated with a Kaiser–Meyer–Olkin measure of sampling adequacy of 0.893 in the left hemisphere and 0.895 in the right hemisphere, which was considered adequate (Kaiser and Rice, 1974). Bartlett’s test of sphericity was significant (left: *x*^2^ [990] = 30,267, *p* < 0.001, right: *x*^2^ [990] = 28,478, *p* < 0.001). Eight PCs were found in the left hemisphere and seven PCs in the right hemisphere, accounting for 63.1 and 59.5% of the common variance, respectively. Details of the PCs are shown in Supplementary Tables 1 and 2. PC1 accounted for 14.23% of the variance in the left hemisphere and 13.52% in the right hemisphere. PC2 accounted for 8.75% in the left hemisphere and 10.47% in the right hemisphere. The AVOI with the highest loadings for PC1 was mostly in the frontal lobe. In contrast, the AVOI with the highest loading for PC2 was clustered around the limbic system to the temporal lobe, such as the hippocampus and the amygdala. For both PCs, higher scores indicated smaller brain volume. For PC3 and the subsequent PCs, the AVOI with high factor loadings tended to be inconsistent between the left and right hemispheres, and anatomical interpretation was considered difficult. Therefore, subsequent analyses were performed only for PC1 and PC2. Figure 2 shows the AVOIs with factor loadings of 0.4 or higher for PC1 and PC2.

### 3.3 Association of PC scores with MMSE scores

**Figure 3:**
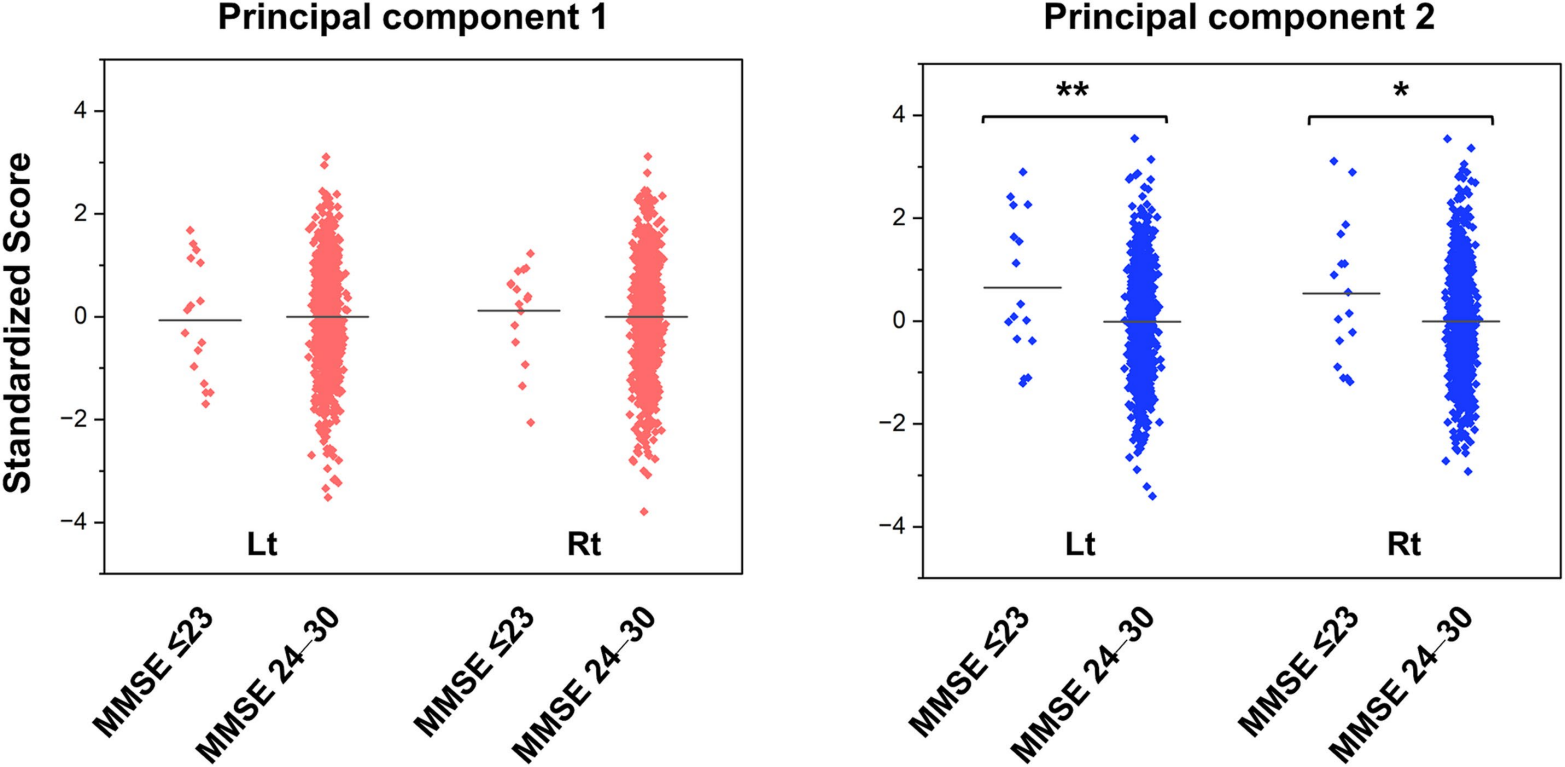
Jitter plots of the principal component scores for the groups with normal cognitive function and cognitive impairment. The PC scores are standardized to a mean of zero and a standard deviation of one. Higher scores indicate smaller brain volume. The horizontal lines in the plots represent the means. The *p* values were calculated by the Student’s *t*-test (* *p* < 0.05, ** *p* < 0.01). MMSE, Mini-Mental State Examination; PC, principal component.

Figure 3 shows the jitter plots of PC scores stratified by MMSE subgroups, and Supplementary Table 3 shows the results of the Student’s *t*-test. As expected, the MMSE scores did not follow a normal distribution (Kolmogorov–Smirnov, *p* < 0.001). No significant differences in PC1 scores were found between the cognitive normal to mild cognitive impairment group (MMSE score: 24–30) and moderate to severe cognitive impairment groups (MMSE score ≤ 23) in the left or right hemispheres. In contrast, the mean PC2 score in the moderate to severe cognitive impairment group was significantly higher than that in the cognitive normal to mild cognitive impairment group in the left hemisphere (*t* = −2.630, *p* = 0.009) and the right hemisphere (*t* = −2.160, *p* = 0.031).

### 3.4 Association of anemia with PC scores and hippocampal volume

**Figure 4:**
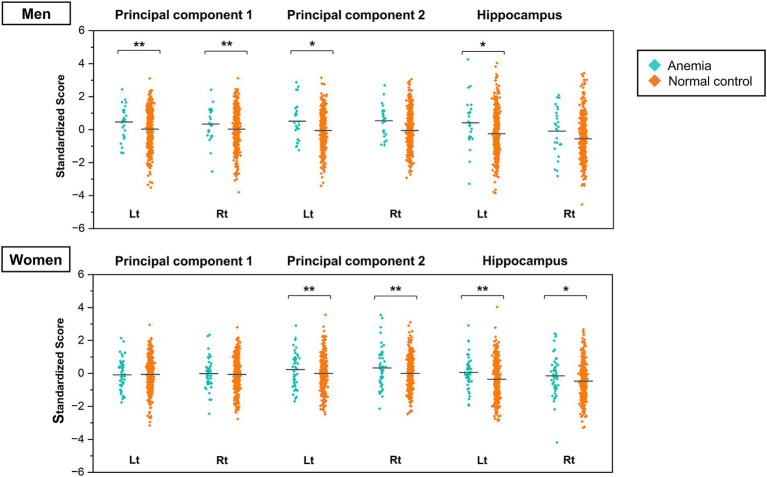
Jitter plots of the principal component scores for anemia and normal control groups stratified by sex. The PC scores are standardized to a mean of zero and a standard deviation of one. Higher scores indicate smaller brain volume. The horizontal lines in the plots represent the means. The *p* values were calculated by the ANCOVA adjusted for participants’ age, BMI, history of hypertension, diabetes mellitus, dyslipidemia, and MRI magnetic field strength (* *p* < 0.05, ** *p* < 0.01). ANCOVA, analysis of covariance; BMI, body mass index; MRI, magnetic resonance imaging; PC, principal component.

The two types of PCs extracted from the PCA were used as dependent variables, and the blood hemoglobin levels classified into the normal control and anemic groups were used as explanatory variables in the ANCOVA. The analyses were performed separately for men and women, with the effects of age, BMI, history of hypertension, diabetes mellitus, dyslipidemia, and MRI magnetic field strength adjusted in the model. The mean and SD of scores in each group are shown in Supplementary Table 4, and the results of the ANCOVA are shown in Figure 4 and Supplementary Table 5. In the male subgroup, PC1 scores were significantly higher in the group with anemia than in the normal control group (left: *F* = 8.455, *p* = 0.004, right: *F* = 8.400, *p* = 0.004). The PC2 scores were higher in the group with anemia in the left hemisphere only (*F* = 3.965, *p* = 0.047). Conversely, in the female subgroup, PC1 scores were not significantly different between the group with anemia and the normal control, while PC2 scores in the group with anemia were significantly higher than in the normal control group (left: *F* = 9.606, *p* = 0.002, right: *F* = 11.237, *p* < 0.001). The ANCOVA with hippocampal volume, the primary anatomical component of PC2, showed significantly higher scores only in the left hemisphere in the male group with anemia (*F* = 4.709, *p* = 0.030) and higher scores in both hemispheres in the female group with anemia (left: *F* = 8.455, *p* = 0.004, right: *F* = 8.400, *p* = 0.004). These findings indicate smaller bilateral PC1 and left PC2 volumes in the male group with anemia and smaller bilateral PC2 volumes in the female group with anemia.

### 3.5 Analyses restricted to 3.0 T scanner MRI data

The present study collected MRI data from 108 participants using the 1.5 T scanner. Therefore, further analyses were limited to data collected on the 3.0 T MRI scanner (n = 921). PCA identified PC1 and PC2, similar to the previous analysis (Supplementary Tables 6, 7). The ANCOVA was also stratified by sex, with participants’ age, BMI, and history of hypertension, diabetes mellitus, and dyslipidemia as covariates. In the male subgroup, there was no significant difference in the left PC2 between the group with anemia and the normal control group. However, the remaining results were consistent with the analysis conducted on the total sample (Supplementary Table 8).

## 4 Discussion

One of the aims of this study was to determine whether the effect of anemia on brain volume is non-selective or site-specific. We measured brain volumes using MRI and summarized the patterns of brain volume using PCA in a large sample of middle-aged and community-dwelling older Japanese adults. The analysis identified PC1, which represented smaller volumes in the frontal lobe, and PC2, which represented smaller volumes in the limbic system and the temporal lobe. Specifically, lower MMSE scores (≤ 23) were significantly associated with higher PC2 scores, which may be influenced by a more severe volume loss in the limbic system. Furthermore, the male group with anemia exhibited markedly elevated PC1 scores compared with the normal control group, whereas the female group with anemia demonstrated significantly higher PC2 scores than the normal control group. These findings indicate a potential association between anemia and smaller volumes in specific brain regions, with evidence suggesting that the distribution of brain volume may vary between sexes.

The primary rationale for using PCA was to identify the prevailing patterns of brain volume in our participants using dimension reduction without focusing on specific brain regions. Brain regions represented by PC1 and PC2 were almost identical in the left and right hemispheres. Similar PCs were also extracted when the analysis was restricted to MRI data from the 3.0 T scanner. This observation demonstrates the consistency of PC1 and PC2 within the same population. However, the interpretation of PCs varies considerably depending on the age, disease background, and other characteristics of the target population. In the present analysis, the PCs should be interpreted with careful consideration of the specific characteristics of Japanese, middle-aged and older participants. The PC1 scores specifically reflected the smaller volumes of various parts of the frontal lobe. Selective frontal lobe atrophy may be present in some degenerative diseases such as frontotemporal dementia (Neary et al., 1988). However, frontotemporal dementia is unlikely to be the primary cause of the PC1 scores as it is less common in healthy populations. Aging is the most significant risk factor for frontal lobe atrophy, and the variations in PC1 scores may indicate individual differences in age-related atrophy (Coffey et al., 1992; Allen et al., 2005). In addition, the absence of a significant association between the PC1 and MMSE scores in the present study may indicate the difficulty of assessing frontal lobe dysfunction, such as executive function, using the MMSE. Conversely, PC2 scores were strongly correlated with smaller limbic system volumes. A significant association was observed between the PC2 and MMSE scores, suggesting that the PC2 may represent the extent of diseases involving limbic atrophy, such as AD. Nevertheless, a smaller limbic system volume, particularly in the hippocampus, cannot be considered a definitive predictor of AD as it can occur in healthy individuals (Barnes et al., 2009). Therefore, the PC2 score should not be regarded as a simple indicator of AD severity. The interpretation of the PCs should be reevaluated using different sample populations in the future to ensure reproducibility, and the inclusion of pathological evidence, such as amyloid positron emission tomography, would also be more beneficial.

The biochemical mechanisms underlying the association between anemia and smaller brain volume remain unclear. Previous studies have speculated that chronic anemia may result in a reduction in the capacity of the blood to carry oxygen, which in turn reduces the oxygen supply to the brain, causing oxidative stress, inflammation, and neurodegeneration (Zlokovic, 2011). However, if anemia-associated hypoxia causes neural damage, it may contribute to a more widespread atrophy of the cerebrum. The present study did not identify an association between anemia and PC1 scores in women, which reflects approximately 14% of the variation in smaller brain volume. Conversely, a correlation was observed between women with anemia and PC2 scores. These findings suggest that anemia does not affect neurons in the cerebrum in a diffuse manner. Most neurodegenerative diseases do not present with diffuse brain atrophy. Instead, they promote focal biochemical damage to neurons triggered by the deposition of abnormal proteins. For example, in the context of AD pathology, amyloid-β or tau proteins begin to accumulate predominantly in the limbic system, where neurodegeneration may progress due to selective vulnerability to elevated local oxidative stress (Droge and Schipper, 2007). Anemia may be specifically implicated in the biochemical mechanisms of certain neurodegenerative diseases rather than in an overall decrease in brain volume.

A further noteworthy finding of this study was the sex-based difference in brain volumes associated with anemia. In individuals aged 65 and older, the anemia prevalence is similar in both sexes, and its etiologies tend to be diverse, including iron deficiency, undernutrition, and chronic inflammatory anemia (Guralnik et al., 2004). Among the participants with anemia in this study, 19.2% of men had macrocytic anemia and 24.5% of women had microcytic anemia, indicating that the underlying cause of anemia may also differ between sexes. A previous study assessed brain cortical thickness in patients with anemia observed a significant reduction across broad cortical regions in women (Park et al., 2016). Another study demonstrated that anemia and elevated red cell distribution width were significantly associated with decreased hippocampal volume, particularly in women (Beydoun et al., 2021). These findings suggest a correlation between anemia and reduced cortical volume in women. Furthermore, the results of this study indicate that anemia in women may be specifically associated with a smaller volume of the hippocampus and limbic system within the cerebral cortex. On the other hand, men were characterized by the presence of macrocytic anemia without any microcytic anemia and by a higher cardiovascular risk burden, such as higher BMI, hypertension, diabetes mellitus, and dyslipidemia. The burden of cardiovascular risk is associated with atrophy of gray matter, including in the hippocampus (Song et al., 2020). Although this study analyzed BMI and medication histories related to hypertension, diabetes mellitus, and dyslipidemia as confounding factors, it is possible that unknown confounding factors, such as heavy alcohol use and nutritional status, might affect the association between anemia and PC1 scores. However, it is premature to assume that anemia is PC1-specific in men because men with anemia also showed significant associations with PC2 scores and hippocampus volume in the left hemisphere. Anemia in men may be more pathologically diverse than in women and may cause smaller volumes in a wide range of brain regions. In this study, serum iron and ferritin levels were not measured, precluding a detailed classification of anemia. Further investigations are required to elucidate the extent to which microcytic anemia, as represented by iron deficiency anemia, contributes to the pathological mechanisms relating anemia and smaller brain volumes in women. In men, the possibility that anemia associated with medical conditions, such as megalocytic anemia and anemia associated with chronic inflammation, may be involved in brain volume reductions should be considered.

A major strength of this study is that the VBM technique was used on data from a large number of participants to measure the volumes of different parts of the brain. The data underwent dimensional reduction using PCA, which enabled the identification of the general patterns of brain volume in statistical analysis. Furthermore, the brain regions represented by PC1 and PC2 were consistent in both hemispheres, allowing easy consideration of the anatomical localization associated with anemia. Although age and cardiovascular risk factors were considered as confounding factors, the present analysis confirmed this association even after adjustment for these factors.

Our study had some limitations. First, this was a cross-sectional study, and the association between anemia and smaller brain volumes did not necessarily imply a causal relationship. Although one prospective study showed that anemia may be a potential risk factor for AD, several studies have indicated that AD pathology may contribute to anemia through chronic inflammation (Faux et al., 2014). The relationship between anemia and smaller limbic system volumes is not necessarily unidirectional. Second, most of the participants in this study were healthy individuals, with only a small number of participants presenting with anemia or cognitive impairment. Additional studies in larger cohorts are needed to verify these findings. Furthermore, the participants tended to be more health-conscious than the general population, which may have led to sample bias. It should be noted that the results of the PCA and interpretation of the PCs varied among sample populations. Third, the number of PCs that could be interpreted anatomically was limited, and each part of the cerebrum was not adequately assessed. For example, the relationship between occipital lobe volume and anemia has not yet been evaluated. A more detailed analysis of the spatial patterns of brain volume distribution using a larger sample size is required. Fourth, the MMSE was the sole measure of cognitive function employed in the present study. A notable proportion of participants tended to achieve high scores on the MMSE. More detailed tests, such as memory recall tests, should have been administered to detect mild cognitive impairment and limbic system dysfunction. Finally, we did not measure AD-specific biomarkers in our participants. For example, including apolipoprotein E gene polymorphism, amyloid positron emission tomography, and cerebrospinal fluid biomarkers might allow us to assess the extent to which reduced limbic system volume in participants is associated with AD.

In conclusion, we identified two main patterns of brain volume distribution using MRI images from the participants in the brain health checkups. The PC2 score, indicating smaller volumes in the limbic system and temporal lobe, was associated with lower MMSE scores and was higher in women with anemia. The findings of this study suggest that anemia may be specifically associated with smaller volumes in the limbic system and provide new evidence on the relationship between anemia and reduced brain volume.

## Data Availability

The raw data supporting the conclusions of this article will be made available by the authors, without undue reservation.
