# Supplementary material for: Anemia-associated smaller brain volume and sex differences: a cross-sectional study of magnetic resonance imaging in brain health checkups
Source: Front Aging Neurosci. 2024 Dec 6;16:1444308. doi: 10.3389/fnagi.2024.1444308 (PMC11659214; doi:10.3389/fnagi.2024.1444308)
Supplement: Supplementary file 1 [file Table_1.docx]

Supplementary Material

Supplementary Table 1. The results of principal component analysis in the left hemisphere for all participants.

| Anatomical regions (Left) | loadings | | | | | | | |
| --- | --- | --- | --- | --- | --- | --- | --- | --- |
|  | PC1 | PC2 | PC3 | PC4 | PC5 | PC6 | PC7 | PC8 |
| Amygdala | 0.19 | 0.54 | 0.06 | 0.13 | –0.03 | 0.09 | 0.57 | 0.19 |
| Angular | 0.10 | 0.24 | 0.15 | 0.03 | 0.69 | 0.01 | –0.12 | 0.09 |
| Calcarine | 0.14 | 0.17 | 0.81 | 0.13 | –0.02 | 0.06 | 0.02 | 0.05 |
| Caudate | 0.07 | 0.02 | 0.06 | –0.09 | 0.13 | 0.03 | –0.02 | 0.72 |
| Cingulum Ant | 0.62 | 0.28 | 0.12 | 0.20 | 0.05 | 0.08 | 0.05 | 0.02 |
| Cingulum Mid | 0.37 | 0.22 | 0.12 | 0.11 | 0.44 | 0.29 | 0.03 | 0.08 |
| Cingulum Post | 0.20 | –0.02 | 0.04 | 0.22 | 0.59 | 0.02 | 0.29 | –0.03 |
| Cuneus | 0.13 | 0.09 | 0.76 | 0.07 | 0.08 | 0.30 | –0.03 | 0.08 |
| Frontal Inf Oper | 0.39 | 0.16 | 0.11 | 0.37 | 0.09 | 0.18 | 0.00 | 0.00 |
| Frontal Inf Orb | 0.63 | 0.09 | 0.10 | 0.28 | 0.18 | 0.12 | 0.27 | –0.02 |
| Frontal Inf Tri | 0.58 | 0.12 | 0.11 | 0.21 | 0.15 | 0.14 | 0.11 | 0.01 |
| Frontal Med Orb | 0.73 | 0.21 | 0.15 | 0.19 | 0.02 | 0.02 | 0.04 | –0.03 |
| Frontal Mid | 0.63 | 0.12 | 0.08 | 0.08 | 0.19 | 0.31 | –0.02 | 0.05 |
| Frontal Mid Orb | 0.74 | –0.01 | 0.14 | 0.03 | 0.09 | 0.02 | 0.10 | 0.03 |
| Frontal Sup | 0.53 | 0.20 | 0.04 | 0.08 | 0.13 | 0.48 | –0.12 | 0.03 |
| Frontal Sup Orb | 0.84 | 0.06 | 0.14 | 0.05 | 0.13 | 0.00 | 0.07 | –0.02 |
| Fusiform | 0.24 | 0.51 | 0.37 | 0.13 | 0.19 | 0.14 | 0.27 | –0.02 |
| Frontal Sup Medial | 0.59 | 0.25 | 0.15 | 0.11 | 0.18 | 0.35 | –0.17 | 0.01 |
| Heschl | 0.25 | 0.08 | 0.21 | 0.81 | 0.04 | 0.06 | 0.15 | –0.05 |
| Hippocampus | 0.11 | 0.42 | 0.17 | 0.12 | 0.03 | –0.01 | 0.72 | 0.10 |
| Insula | 0.49 | 0.20 | 0.04 | 0.55 | 0.11 | 0.04 | 0.19 | 0.18 |
| Lingual | 0.15 | 0.25 | 0.74 | 0.11 | 0.06 | 0.11 | 0.12 | 0.02 |
| Occipital Inf | 0.18 | 0.11 | 0.59 | 0.12 | 0.22 | –0.14 | 0.12 | –0.07 |
| Occipital Mid | 0.26 | –0.02 | 0.65 | 0.07 | 0.38 | 0.00 | 0.08 | –0.01 |
| Occipital Sup | 0.10 | –0.10 | 0.70 | 0.13 | 0.10 | 0.27 | 0.06 | 0.06 |
| Olfactory | 0.56 | 0.24 | 0.09 | 0.27 | 0.07 | –0.02 | 0.24 | 0.10 |
| Pallidum | –0.04 | 0.04 | 0.01 | 0.02 | –0.01 | 0.04 | 0.08 | 0.82 |
| Paracentral lobule | –0.01 | 0.02 | 0.14 | –0.05 | 0.09 | 0.79 | 0.14 | 0.07 |
| Parahippocampal | 0.16 | 0.64 | 0.18 | 0.04 | 0.05 | 0.08 | 0.56 | 0.07 |
| Parietal Inf | 0.14 | 0.21 | 0.09 | 0.25 | 0.69 | 0.26 | –0.04 | 0.10 |
| Parietal Sup | 0.10 | –0.11 | 0.11 | 0.03 | 0.55 | 0.30 | 0.10 | 0.05 |
| Postcentral | 0.03 | 0.16 | 0.12 | 0.33 | 0.32 | 0.56 | 0.17 | 0.06 |
| Precentral | 0.17 | 0.03 | 0.10 | 0.10 | 0.11 | 0.76 | 0.17 | 0.02 |
| Precuneus | 0.19 | –0.01 | 0.27 | 0.12 | 0.63 | 0.39 | 0.13 | 0.01 |
| Putamen | 0.03 | 0.06 | 0.03 | 0.05 | 0.03 | 0.07 | 0.17 | 0.88 |
| Rectus | 0.79 | 0.13 | 0.13 | 0.11 | 0.07 | 0.02 | 0.09 | 0.00 |
| Rolandic Oper | 0.29 | 0.09 | 0.19 | 0.82 | 0.11 | 0.06 | 0.10 | –0.04 |
| Supp Motor Area | 0.30 | 0.16 | 0.11 | –0.01 | 0.18 | 0.70 | –0.13 | 0.02 |
| Supramarginal | 0.12 | 0.22 | 0.08 | 0.61 | 0.35 | 0.00 | –0.03 | 0.00 |
| Temporal Inf | 0.23 | 0.67 | 0.13 | 0.22 | 0.23 | 0.04 | –0.01 | 0.08 |
| Temporal Mid | 0.24 | 0.59 | 0.13 | 0.19 | 0.44 | –0.02 | 0.05 | 0.02 |
| Temporal Pole Mid | 0.19 | 0.79 | 0.03 | 0.12 | –0.03 | 0.13 | 0.01 | 0.01 |
| Temporal Pole Sup | 0.32 | 0.70 | 0.05 | 0.28 | 0.03 | 0.10 | 0.14 | 0.01 |
| Temporal Sup | 0.21 | 0.31 | 0.11 | 0.76 | 0.13 | 0.05 | 0.06 | –0.01 |
| Thalamus | 0.12 | –0.04 | 0.06 | 0.09 | 0.10 | 0.14 | 0.56 | 0.08 |
|  |  |  |  |  |  |  |  |  |
| Percentage of variance (%) | 14.23 | 8.75 | 8.32 | 8.01 | 7.15 | 7.14 | 4.78 | 4.76 |
| Cumulative percentage of variance (%) | 14.2 | 23 | 31.3 | 39.3 | 46.5 | 53.6 | 58.4 | 63.1 |

PC, principal component.

**Supplementary Table 2. The results of principal component analysis in the right hemisphere for all participants.**

| Anatomical regions (Right) | loadings | | | | | | |
| --- | --- | --- | --- | --- | --- | --- | --- |
|  | PC1 | PC2 | PC3 | PC4 | PC5 | PC6 | PC7 |
| Amygdala | 0.16 | 0.77 | 0.08 | 0.07 | 0.06 | 0.00 | 0.22 |
| Angular | 0.20 | 0.10 | 0.07 | 0.13 | 0.01 | 0.76 | 0.07 |
| Calcarine | 0.08 | 0.23 | 0.04 | 0.72 | 0.10 | –0.08 | 0.00 |
| Caudate | –0.01 | 0.03 | 0.15 | 0.08 | –0.16 | 0.09 | 0.63 |
| Cingulum Ant | 0.55 | 0.24 | 0.13 | 0.11 | 0.01 | 0.08 | –0.06 |
| Cingulum Mid | 0.38 | 0.20 | 0.44 | 0.17 | 0.03 | 0.35 | 0.02 |
| Cingulum Post | 0.19 | 0.09 | 0.01 | 0.12 | 0.25 | 0.44 | 0.01 |
| Cuneus | 0.11 | 0.08 | 0.27 | 0.75 | 0.10 | 0.09 | 0.00 |
| Frontal Inf Oper | 0.41 | 0.06 | 0.22 | 0.01 | 0.49 | 0.14 | 0.00 |
| Frontal Inf Orb | 0.68 | 0.18 | 0.11 | 0.08 | 0.23 | 0.11 | –0.03 |
| Frontal Inf Tri | 0.57 | 0.06 | 0.16 | 0.06 | 0.31 | 0.07 | –0.02 |
| Frontal Med Orb | 0.74 | 0.18 | 0.11 | 0.14 | 0.15 | 0.07 | 0.02 |
| Frontal Mid | 0.78 | 0.11 | 0.06 | 0.12 | 0.03 | 0.07 | 0.02 |
| Frontal Mid Orb | 0.62 | 0.10 | 0.32 | 0.12 | 0.12 | 0.16 | 0.00 |
| Frontal Sup | 0.49 | 0.08 | 0.37 | 0.07 | 0.13 | 0.20 | 0.00 |
| Frontal Sup Orb | 0.85 | 0.09 | 0.06 | 0.15 | 0.06 | 0.12 | 0.03 |
| Fusiform | 0.48 | 0.06 | 0.56 | 0.08 | 0.16 | 0.15 | 0.03 |
| Frontal Sup Medial | 0.23 | 0.57 | 0.22 | 0.27 | 0.17 | 0.19 | 0.01 |
| Heschl | 0.17 | 0.22 | 0.10 | 0.22 | 0.78 | 0.06 | –0.03 |
| Hippocampus | 0.08 | 0.67 | 0.02 | 0.25 | 0.08 | –0.01 | 0.27 |
| Insula | 0.51 | 0.34 | 0.05 | 0.10 | 0.54 | 0.13 | 0.13 |
| Lingual | 0.15 | 0.31 | 0.11 | 0.68 | 0.10 | –0.01 | 0.00 |
| Occipital Inf | 0.16 | 0.09 | –0.06 | 0.64 | 0.09 | 0.23 | 0.03 |
| Occipital Mid | 0.25 | –0.02 | 0.06 | 0.66 | 0.07 | 0.39 | 0.09 |
| Occipital Sup | 0.12 | –0.04 | 0.24 | 0.72 | 0.11 | 0.18 | 0.10 |
| Olfactory | 0.53 | 0.42 | –0.07 | 0.09 | 0.15 | 0.11 | 0.12 |
| Pallidum | –0.04 | 0.03 | 0.03 | –0.01 | 0.06 | 0.02 | 0.84 |
| Paracentral lobule | 0.04 | 0.07 | 0.77 | 0.14 | –0.06 | 0.05 | 0.10 |
| Parahippocampal | 0.17 | 0.83 | 0.11 | 0.15 | 0.06 | 0.03 | 0.15 |
| Parietal Inf | 0.05 | 0.13 | 0.25 | 0.02 | 0.11 | 0.72 | 0.04 |
| Parietal Sup | 0.08 | –0.11 | 0.39 | 0.14 | 0.07 | 0.51 | 0.07 |
| Postcentral | 0.04 | 0.10 | 0.61 | 0.06 | 0.37 | 0.21 | 0.05 |
| Precentral | 0.17 | 0.08 | 0.74 | 0.11 | 0.14 | –0.02 | 0.15 |
| Precuneus | 0.20 | 0.09 | 0.39 | 0.34 | 0.14 | 0.52 | –0.02 |
| Putamen | 0.08 | 0.16 | 0.05 | 0.03 | 0.14 | –0.02 | 0.86 |
| Rectus | 0.76 | 0.14 | 0.06 | 0.12 | 0.13 | 0.08 | 0.06 |
| Rolandic Oper | 0.25 | 0.22 | 0.07 | 0.18 | 0.79 | 0.12 | –0.03 |
| Supp Motor Area | 0.27 | 0.09 | 0.74 | 0.08 | –0.02 | 0.17 | 0.01 |
| Supramarginal | 0.09 | 0.21 | 0.01 | 0.06 | 0.53 | 0.50 | –0.02 |
| Temporal Inf | 0.21 | 0.62 | –0.05 | 0.11 | 0.19 | 0.36 | –0.10 |
| Temporal Mid | 0.23 | 0.50 | –0.01 | 0.22 | 0.23 | 0.46 | 0.00 |
| Temporal Pole Mid | 0.14 | 0.69 | 0.08 | –0.05 | 0.22 | 0.06 | –0.13 |
| Temporal Pole Sup | 0.23 | 0.68 | 0.12 | 0.01 | 0.28 | 0.07 | –0.08 |
| Temporal Sup | 0.20 | 0.38 | 0.05 | 0.11 | 0.58 | 0.27 | 0.01 |
| Thalamus | 0.11 | 0.12 | 0.11 | 0.10 | 0.27 | 0.03 | 0.19 |
|  |  |  |  |  |  |  |  |
| Percentage of variance (%) | 13.52 | 10.47 | 8.05 | 8.04 | 7.46 | 7.07 | 4.84 |
| Cumulative percentage of variance (%) | 13.5 | 24 | 32 | 40.1 | 47.5 | 54.6 | 59.5 |

PC, principal component.

|  | **Mean** | | **Df** | ***t*** | ***p*** |
| --- | --- | --- | --- | --- | --- |
|  | MMSE score 24–30 (n = 1,013) | MMSE score ≤23 (n = 16) |  |  |  |
| Lt PC1 | 0.001 | –0.072 | 1,027 | 0.289 | 0.772 |
| Rt PC1 | –0.002 | 0.117 | 1,027 | –0.498 | 0.619 |
| Lt PC2 | –0.010 | 0.649 | 1,027 | –2.630 | **0.009** |
| Rt PC2 | –0.008 | 0.534 | 1,027 | –2.160 | **0.031** |

**Supplementary Table 3.** **The result of the Student’s t-test.**

Df, degrees of freedom; MMSE, Mini-Mental State Examination; PC, principal component.

**Supplementary Table 4. Mean and standard deviation of principal component scores and hippocampal volume scores for each group.**

| **Standardized score, mean (SD)** | | **Men** | **Women** |
| --- | --- | --- | --- |
| Lt PC1 | Anemia | 0.396 (0.98) | –0.045 (0.99) |
|  | NC | –0.019 (1.00) | 0.005 (1.00) |
| Rt PC1 | Anemia | 0.316 (0.98) | 0.026 (0.953) |
|  | NC | –0.016 (1.00) | –0.003 (1.01) |
| Lt PC2 | Anemia | 0.496 (1.28) | 0.318 (1.10) |
|  | NC | –0.024 (0.98) | –0.037 (0.98) |
| Rt PC2 | Anemia | 0.545 (0.89) | 0.326 (1.27) |
|  | NC | –0.027 (1.00) | –0.038 (0.958) |
| Lt hippocampus | Anemia | 0.410 (1.49) | 0.062 (0.942) |
|  | NC | –0.255 (1.24) | –0.358 (1.04) |
| Rt hippocampus | Anemia | –0.091 (1.43) | –0.149 (1.19) |
|  | NC | –0.553 (1.23) | –0.467 (1.05) |

NC, normal control; PC, principal component; SD, standard deviation.

**Supplementary Table 5. Analysis of covariance stratified by sex.**

| **Source** | **Dependent variable** | **Sum of squares** | **Df** | **Mean square** | **F** | ***p* value** |
| --- | --- | --- | --- | --- | --- | --- |
| *Male participants* Anemia = 26  NC = 531 | Lt PC1 | 8.029 | 1 | 8.029 | 8.455 | 0.004** |
|  | Rt PC1 | 7.705 | 1 | 7.705 | 8.200 | 0.004** |
|  | Lt PC2 | 3.889 | 1 | 3.889 | 3.965 | 0.047* |
|  | Rt PC2 | 1.467 | 1 | 1.467 | 1.634 | 0.202 |
|  | Lt hippocampus | 6.994 | 1 | 6.994 | 4.709 | 0.030* |
|  | Rt hippocampus | 1.664 | 1 | 1.664 | 1.163 | 0.281 |
| *Female participants* Anemia = 49 NC = 423 | Lt PC1 | 0.172 | 1 | 0.172 | 0.190 | 0.664 |
|  | Rt PC1 | 0.000 | 1 | 0.000 | 0.000 | 0.998 |
|  | Lt PC2 | 8.321 | 1 | 8.321 | 9.606 | 0.002** |
|  | Rt PC2 | 9.381 | 1 | 9.381 | 11.237 | < 0.001** |
|  | Lt hippocampus | 7.878 | 1 | 7.878 | 8.130 | 0.005** |
|  | Rt hippocampus | 4.880 | 1 | 4.880 | 4.847 | 0.028* |

All models were adjusted for participants’ age, BMI, history of hypertension, diabetes mellitus, dyslipidemia, and MRI magnetic field strength (* *p* < 0.05, ** *p* < 0.01). BMI, body mass index; Df, degrees of freedom; NC, normal control; PC, principal component.

**Supplementary Table 6. The results of principal component analysis in the left hemisphere restricted to 3.0 T MRI scanner data.**

| Anatomical regions (Left) | loadings | | | | | | |
| --- | --- | --- | --- | --- | --- | --- | --- |
|  | PC1 | PC2 | PC3 | PC4 | PC5 | PC6 | PC7 |
| Amygdala | 0.17 | 0.75 | 0.06 | 0.16 | 0.05 | –0.03 | 0.21 |
| Angular | 0.08 | 0.14 | 0.11 | –0.01 | 0.04 | 0.73 | 0.03 |
| Calcarine | 0.15 | 0.20 | 0.79 | 0.13 | 0.05 | 0.00 | 0.00 |
| Caudate | 0.05 | 0.03 | 0.07 | –0.10 | 0.06 | 0.15 | 0.70 |
| Cingulum Ant | 0.57 | 0.29 | 0.13 | 0.21 | 0.16 | 0.04 | 0.03 |
| Cingulum Mid | 0.31 | 0.22 | 0.15 | 0.13 | 0.36 | 0.41 | 0.09 |
| Cingulum Post | 0.23 | 0.07 | 0.05 | 0.24 | –0.02 | 0.57 | 0.01 |
| Cuneus | 0.11 | 0.09 | 0.76 | 0.08 | 0.30 | 0.10 | 0.06 |
| Frontal Inf Oper | 0.39 | 0.16 | 0.14 | 0.34 | 0.17 | 0.10 | 0.01 |
| Frontal Inf Orb | 0.65 | 0.20 | 0.11 | 0.28 | 0.10 | 0.18 | –0.02 |
| Frontal Inf Tri | 0.59 | 0.14 | 0.14 | 0.19 | 0.12 | 0.15 | 0.00 |
| Frontal Med Orb | 0.71 | 0.25 | 0.14 | 0.19 | 0.08 | 0.01 | –0.02 |
| Frontal Mid | 0.59 | 0.08 | 0.10 | 0.07 | 0.37 | 0.20 | 0.05 |
| Frontal Mid Orb | 0.75 | 0.03 | 0.14 | 0.02 | 0.06 | 0.09 | 0.01 |
| Frontal Sup | 0.50 | 0.12 | 0.04 | 0.05 | 0.52 | 0.14 | 0.01 |
| Frontal Sup Orb | 0.54 | 0.15 | 0.17 | 0.09 | 0.45 | 0.21 | –0.01 |
| Fusiform | 0.83 | 0.10 | 0.15 | 0.05 | 0.09 | 0.14 | –0.01 |
| Frontal Sup Medial | 0.23 | 0.58 | 0.34 | 0.12 | 0.16 | 0.19 | –0.04 |
| Heschl | 0.25 | 0.15 | 0.21 | 0.81 | 0.06 | 0.05 | –0.03 |
| Hippocampus | 0.16 | 0.71 | 0.15 | 0.13 | –0.11 | 0.03 | 0.12 |
| Insula | 0.51 | 0.26 | 0.04 | 0.53 | 0.04 | 0.13 | 0.16 |
| Lingual | 0.17 | 0.30 | 0.72 | 0.10 | 0.08 | 0.07 | –0.02 |
| Occipital Inf | 0.21 | 0.19 | 0.54 | 0.10 | –0.14 | 0.22 | –0.09 |
| Occipital Mid | 0.26 | 0.03 | 0.64 | 0.05 | –0.01 | 0.38 | 0.00 |
| Occipital Sup | 0.10 | –0.04 | 0.72 | 0.15 | 0.23 | 0.09 | 0.09 |
| Olfactory | 0.53 | 0.34 | 0.08 | 0.30 | 0.02 | 0.06 | 0.13 |
| Pallidum | –0.02 | 0.07 | –0.03 | 0.04 | –0.01 | –0.01 | 0.80 |
| Paracentral lobule | –0.01 | 0.07 | 0.10 | –0.02 | 0.79 | 0.04 | 0.02 |
| Parahippocampal | 0.15 | 0.83 | 0.16 | 0.04 | 0.07 | 0.06 | 0.09 |
| Parietal Inf | 0.12 | 0.15 | 0.09 | 0.23 | 0.29 | 0.69 | 0.10 |
| Parietal Sup | 0.09 | –0.10 | 0.16 | 0.06 | 0.28 | 0.50 | 0.13 |
| Postcentral | 0.08 | 0.20 | 0.10 | 0.36 | 0.50 | 0.31 | 0.05 |
| Precentral | 0.21 | 0.07 | 0.06 | 0.11 | 0.71 | 0.06 | 0.02 |
| Precuneus | 0.17 | 0.02 | 0.29 | 0.15 | 0.38 | 0.59 | 0.05 |
| Putamen | 0.07 | 0.14 | –0.01 | 0.05 | 0.02 | 0.04 | 0.88 |
| Rectus | 0.77 | 0.19 | 0.13 | 0.12 | 0.11 | 0.06 | 0.02 |
| Rolandic Oper | 0.29 | 0.13 | 0.20 | 0.82 | 0.07 | 0.12 | –0.02 |
| Supp Motor Area | 0.24 | 0.07 | 0.10 | –0.02 | 0.74 | 0.18 | –0.01 |
| Supramarginal | 0.11 | 0.19 | 0.06 | 0.59 | 0.03 | 0.38 | –0.02 |
| Temporal Inf | 0.21 | 0.59 | 0.10 | 0.20 | 0.10 | 0.31 | –0.01 |
| Temporal Mid | 0.23 | 0.54 | 0.08 | 0.15 | 0.01 | 0.50 | –0.06 |
| Temporal Pole Mid | 0.13 | 0.71 | 0.04 | 0.10 | 0.23 | 0.03 | –0.03 |
| Temporal Pole Sup | 0.26 | 0.69 | 0.05 | 0.28 | 0.18 | 0.06 | –0.02 |
| Temporal Sup | 0.20 | 0.31 | 0.10 | 0.75 | 0.08 | 0.17 | –0.02 |
| Thalamus | 0.25 | 0.22 | 0.00 | 0.05 | –0.02 | 0.11 | 0.10 |
|  |  |  |  |  |  |  |  |
| Percentage of variance (%) | 13.55 | 10.62 | 8.07 | 7.87 | 7.58 | 7.32 | 4.68 |
| Cumulative percentage of variance (%) | 13.6 | 24.2 | 32.2 | 40.1 | 47.7 | 55.0 | 59.7 |

The Bartlett’s test of sphericity was significant (*x*^2^ (990) = 26,546, *p* < 0.001). PC, principal component.

**Supplementary Table 7. The results of principal component analysis in the right hemisphere restricted to 3.0 T MRI scanner data.**

| Anatomical regions (Right) | loadings | | | | | | |
| --- | --- | --- | --- | --- | --- | --- | --- |
|  | PC1 | PC2 | PC3 | PC4 | PC5 | PC6 | PC7 |
| Amygdala | 0.17 | 0.80 | 0.04 | 0.05 | 0.07 | 0.02 | 0.14 |
| Angular | 0.19 | 0.13 | 0.05 | 0.11 | 0.01 | 0.76 | 0.04 |
| Calcarine | 0.08 | 0.23 | 0.04 | 0.73 | 0.11 | –0.11 | 0.02 |
| Caudate | –0.01 | 0.02 | 0.14 | 0.07 | –0.18 | 0.09 | 0.62 |
| Cingulum Ant | 0.53 | 0.25 | 0.18 | 0.11 | 0.00 | 0.08 | –0.04 |
| Cingulum Mid | 0.34 | 0.21 | 0.50 | 0.17 | 0.03 | 0.32 | 0.05 |
| Cingulum Post | 0.19 | 0.11 | 0.02 | 0.10 | 0.23 | 0.44 | 0.00 |
| Cuneus | 0.11 | 0.07 | 0.27 | 0.75 | 0.12 | 0.10 | –0.02 |
| Frontal Inf Oper | 0.39 | 0.07 | 0.23 | 0.02 | 0.51 | 0.14 | 0.02 |
| Frontal Inf Orb | 0.66 | 0.19 | 0.15 | 0.10 | 0.24 | 0.10 | –0.03 |
| Frontal Inf Tri | 0.55 | 0.05 | 0.15 | 0.07 | 0.33 | 0.06 | –0.03 |
| Frontal Med Orb | 0.74 | 0.19 | 0.10 | 0.15 | 0.15 | 0.05 | 0.03 |
| Frontal Mid | 0.77 | 0.12 | 0.08 | 0.12 | 0.05 | 0.07 | 0.00 |
| Frontal Mid Orb | 0.60 | 0.10 | 0.34 | 0.13 | 0.14 | 0.16 | –0.01 |
| Frontal Sup | 0.48 | 0.07 | 0.38 | 0.07 | 0.14 | 0.18 | 0.03 |
| Frontal Sup Orb | 0.85 | 0.09 | 0.08 | 0.15 | 0.06 | 0.12 | 0.03 |
| Fusiform | 0.47 | 0.06 | 0.56 | 0.07 | 0.19 | 0.16 | 0.05 |
| Frontal Sup Medial | 0.20 | 0.59 | 0.25 | 0.26 | 0.16 | 0.17 | 0.00 |
| Heschl | 0.17 | 0.23 | 0.07 | 0.22 | 0.78 | 0.03 | –0.04 |
| Hippocampus | 0.14 | 0.72 | –0.04 | 0.19 | 0.05 | 0.03 | 0.14 |
| Insula | 0.50 | 0.35 | 0.07 | 0.11 | 0.54 | 0.12 | 0.12 |
| Lingual | 0.15 | 0.32 | 0.13 | 0.68 | 0.10 | –0.04 | 0.02 |
| Occipital Inf | 0.17 | 0.11 | –0.09 | 0.64 | 0.07 | 0.24 | 0.01 |
| Occipital Mid | 0.27 | 0.01 | 0.01 | 0.64 | 0.06 | 0.43 | 0.03 |
| Occipital Sup | 0.14 | –0.03 | 0.17 | 0.71 | 0.12 | 0.22 | 0.03 |
| Olfactory | 0.52 | 0.44 | –0.07 | 0.09 | 0.15 | 0.11 | 0.11 |
| Pallidum | –0.02 | 0.01 | –0.01 | –0.03 | 0.07 | 0.02 | 0.85 |
| Paracentral lobule | 0.04 | 0.06 | 0.77 | 0.13 | –0.09 | 0.04 | 0.06 |
| Parahippocampal | 0.17 | 0.85 | 0.08 | 0.11 | 0.05 | 0.05 | 0.07 |
| Parietal Inf | 0.02 | 0.14 | 0.26 | 0.02 | 0.10 | 0.70 | 0.04 |
| Parietal Sup | 0.07 | –0.11 | 0.31 | 0.12 | 0.09 | 0.53 | 0.03 |
| Postcentral | 0.06 | 0.09 | 0.57 | 0.05 | 0.38 | 0.21 | 0.03 |
| Precentral | 0.21 | 0.08 | 0.71 | 0.05 | 0.17 | 0.00 | 0.06 |
| Precuneus | 0.17 | 0.10 | 0.41 | 0.37 | 0.17 | 0.48 | 0.02 |
| Putamen | 0.11 | 0.15 | 0.03 | 0.02 | 0.14 | –0.02 | 0.88 |
| Rectus | 0.77 | 0.15 | 0.08 | 0.13 | 0.13 | 0.07 | 0.07 |
| Rolandic Oper | 0.25 | 0.23 | 0.08 | 0.18 | 0.78 | 0.10 | –0.02 |
| Supp Motor Area | 0.25 | 0.06 | 0.75 | 0.07 | –0.01 | 0.16 | 0.01 |
| Supramarginal | 0.06 | 0.21 | 0.04 | 0.07 | 0.52 | 0.50 | 0.02 |
| Temporal Inf | 0.17 | 0.61 | 0.00 | 0.13 | 0.21 | 0.34 | –0.06 |
| Temporal Mid | 0.23 | 0.51 | –0.03 | 0.21 | 0.23 | 0.46 | –0.01 |
| Temporal Pole Mid | 0.08 | 0.66 | 0.16 | –0.01 | 0.26 | 0.02 | –0.07 |
| Temporal Pole Sup | 0.18 | 0.67 | 0.16 | 0.03 | 0.31 | 0.03 | –0.05 |
| Temporal Sup | 0.19 | 0.38 | 0.04 | 0.13 | 0.57 | 0.26 | 0.01 |
| Thalamus | 0.16 | 0.15 | 0.08 | 0.05 | 0.23 | 0.10 | 0.06 |
|  |  |  |  |  |  |  |  |
| Percentage of variance (%) | 13.01 | 10.88 | 8.00 | 7.89 | 7.62 | 7.01 | 4.44 |
| Cumulative percentage of variance (%) | 13.0 | 23.9 | 31.9 | 39.8 | 47.4 | 44.4 | 58.9 |

The Bartlett’s test of sphericity was significant (*x*^2^ (990) = 25,115, *p* < 0.001). PC, principal component.

**Supplementary Table 8.** **The results of the analysis of covariance stratified by sex restricted to 3.0 T MRI scanner data.**

| **Source** | **Dependent variable** | **Sum of squares** | **Df** | **Mean square** | **F** | ***p* value** |
| --- | --- | --- | --- | --- | --- | --- |
| *Male participants* Anemia = 22  NC = 473 | Lt PC1 | 4.910 | 1 | 4.910 | 5.031 | 0.025* |
|  | Rt PC1 | 5.605 | 1 | 5.605 | 5.880 | 0.016* |
|  | Lt PC2 | 2.979 | 1 | 2.979 | 3.007 | 0.084 |
|  | Rt PC2 | 1.739 | 1 | 1.739 | 1.956 | 0.163 |
|  | Lt hippocampus | 6.628 | 1 | 6.628 | 4.526 | 0.034* |
|  | Rt hippocampus | 1.148 | 1 | 1.148 | 0.796 | 0.373 |
| *Female participants* Anemia = 45 NC = 381 | Lt PC1 | 0.214 | 1 | 0.214 | 0.216 | 0.643 |
|  | Rt PC1 | 0.000 | 1 | 0.000 | 0.000 | 0.981 |
|  | Lt PC2 | 8.947 | 1 | 8.947 | 9.606 | 0.001** |
|  | Rt PC2 | 12.183 | 1 | 12.183 | 14.915 | < 0.001** |
|  | Lt hippocampus | 7.559 | 1 | 7.559 | 7.605 | 0.006** |
|  | Rt hippocampus | 6.617 | 1 | 6.617 | 46.447 | 0.011* |

All models were adjusted for participants’ age, BMI, history of hypertension, diabetes mellitus, and dyslipidemia (* *p* < 0.05, ** *p* < 0.01). Df, degrees of freedom; MRI, magnetic resonance imaging; NC, normal control; PC, principal component.
